# Supplementary material for: Surface frustration re-patterning underlies the structural landscape and evolvability of fungal orphan candidate effectors
Source: Nat Commun. 2023 Aug 28;14:5244. doi: 10.1038/s41467-023-40949-9 (PMC10462633; doi:10.1038/s41467-023-40949-9)
Supplement: Supplementary file 1 — Supplementary Information [file 41467_2023_40949_MOESM1_ESM.pdf]

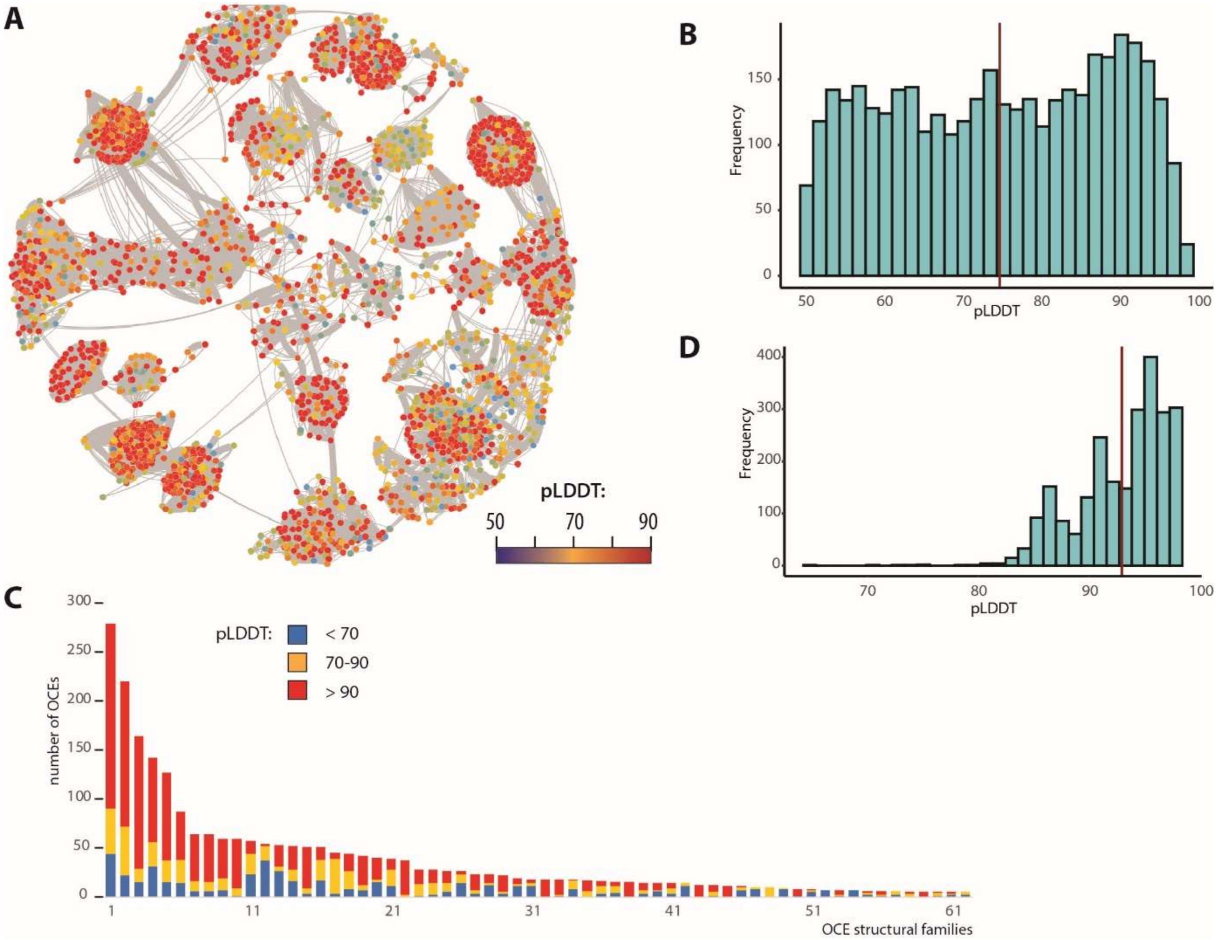

Supplementary Fig. 1. Distribution of pLDDT values across predicted OCE structures. (A) Structural similarity network with pLDDT values mapped on nodes. **(B)** Distribution of pLDDT values among the 3911 OCEs included in our similarity network analysis. **(C)** Number of OCEs with in the three pLDDT values categories in each of the 62 OCE families. **(D)** Distribution of pLDDT values in the major OCE families analyzed in details in this work (see **Source Data** for a list). The red lines in (B) and (D) show mean pLDDT value.

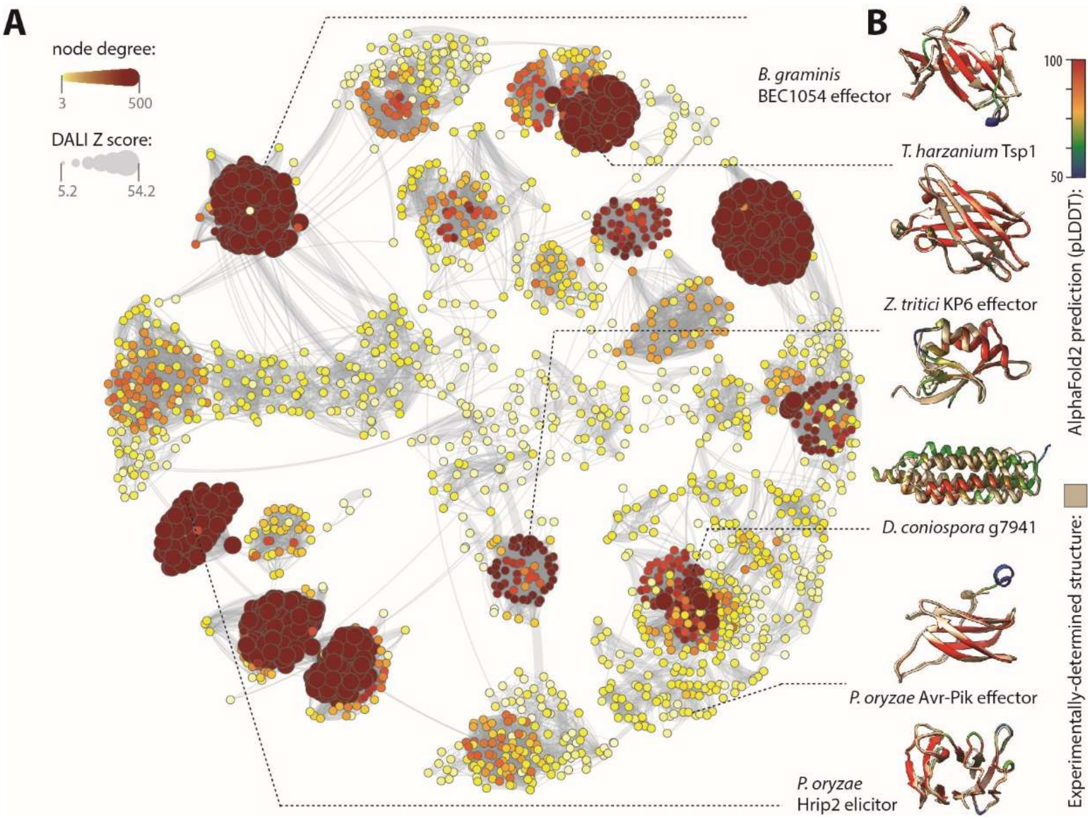

Supplementary Fig. 2. Structural relationship between orphan candidate effectors in 20 fungal genomes. (A) The structural landscape of fungal orphan candidate effectors shown as a similarity network. Vertices are OCE predicted structures colored and sized according to their number of edges which correspond to pairwise Dali Z-scores. Most of the broadly conserved fold covered the full length of OCEs. Exceptions included the short helical folds (1-helix and 3-helix) which were associated with other structural features in some OCEs, reducing the overall structural similarity for proteins harboring these folds **(B)** OCEs of the network for which an experimentally-determined structure is available. Six effectors from the network had experimentally determined structures: *Blumeria graminis* ribonuclease (RNase)-like fold (RALPH) CSEP0064/BEC1054 (6fmb)[1], *Trichoderma* Tsp1 (7esw)[2], *Zymoseptoria tritici* KP6 killer toxin (6qpk)[3], *Drechmeria coniospora* g7941 (6zpp), and *Pyricularia oryzae* Avr-Pik avirulence effector (6fud)[4] and Hrip2 elicitor (5fid)[5]. Predicted structures colored according to pLDDT score are superimposed with experimentally determined structures (tan color). Dotted lines point towards the corresponding vertices in the network.

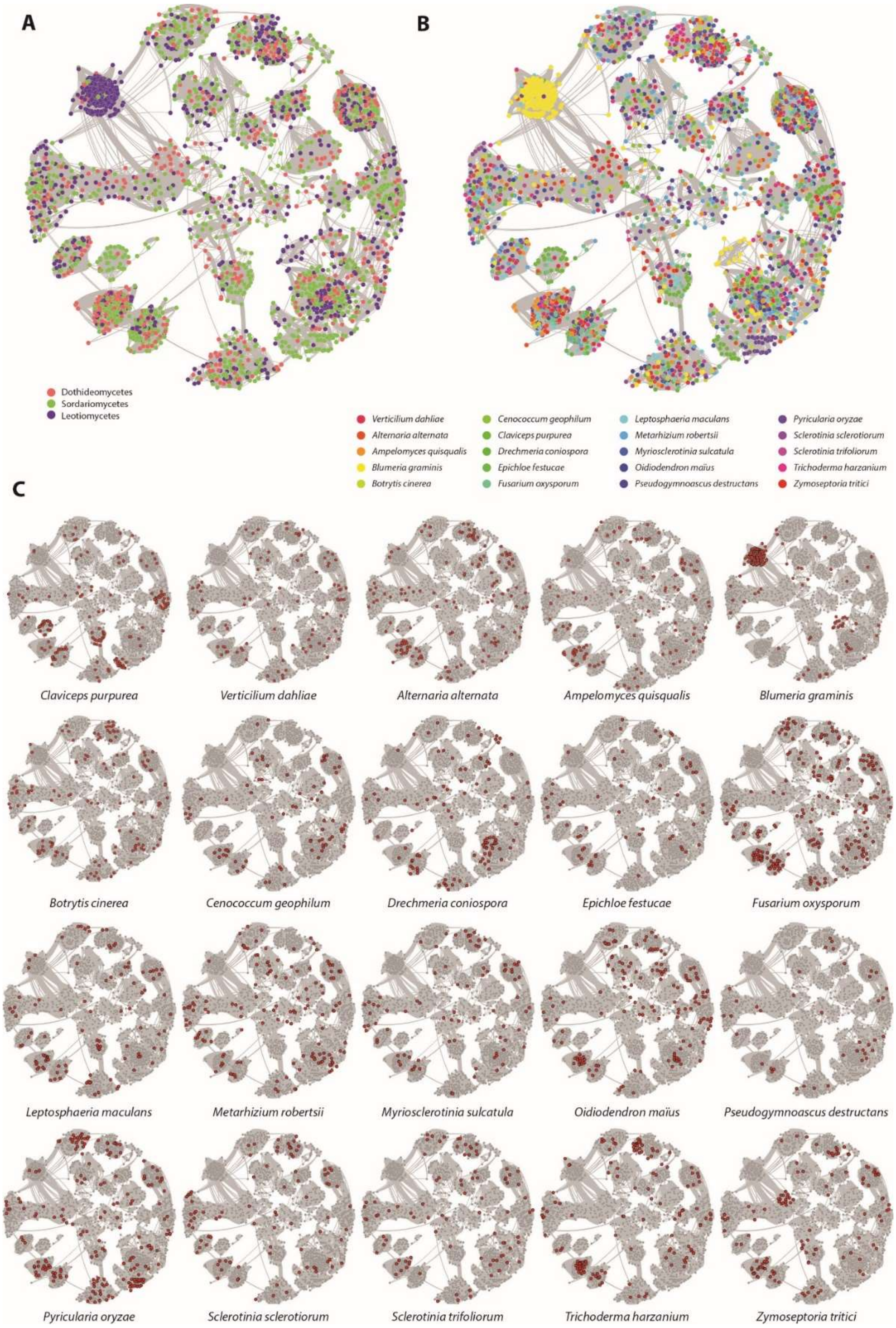

Supplementary Fig. 3. Lineage and species representation across the OCE structural similarity network. (A) Mapping of the three major fungal lineages onto the structural similarity network, with nodes colored according to the lineage they belong to. **(B)** Mapping of the twenty fungal species onto the structural similarity network, with nodes colored according to the species they belong to. **(C)** Structural similarity network with nodes corresponding to each of the twenty species highlighted.

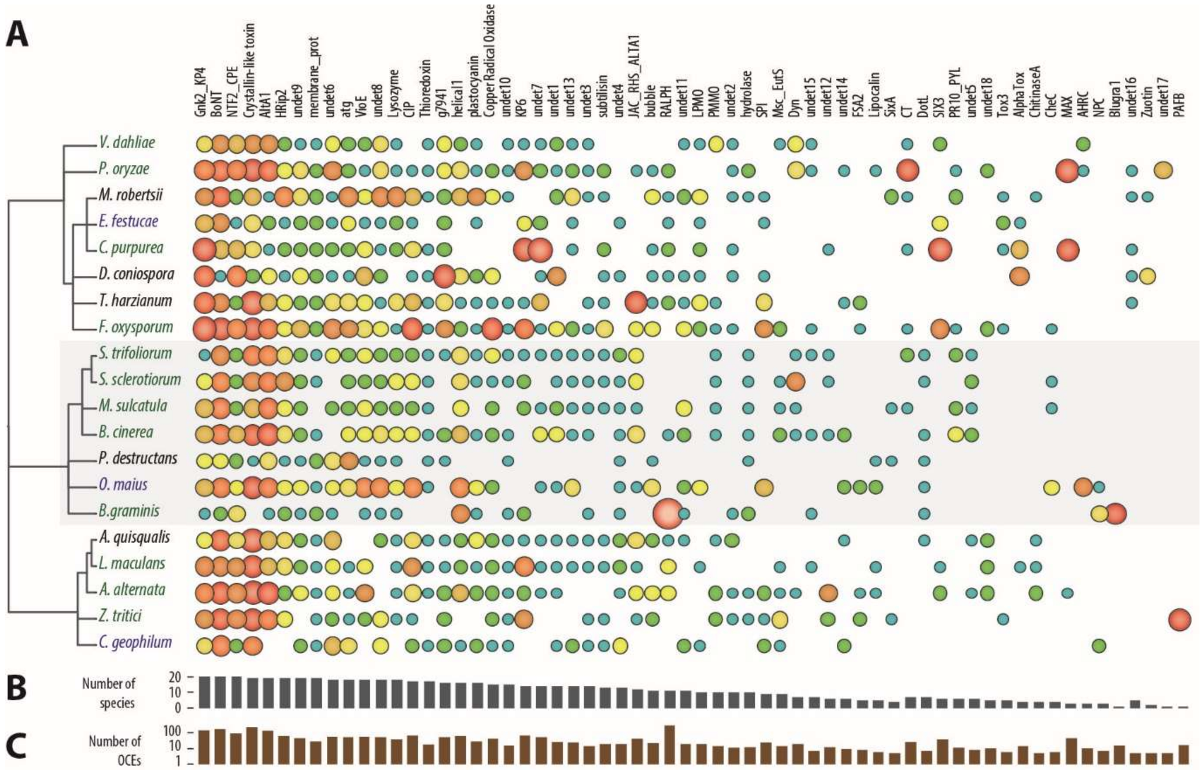

Supplementary Fig. 4. Species distribution of the 62 major OCE structural groups. (A) Number of OCEs from each of the 62 major family in the 20 fungal species analyzed (see also Table S4). Number of occurrences ranges from 1 (light blue) to 262 (RALPH in *B. graminis*, red). **(B)** Number of species containing at least one member of the OCE family. **(C)** total number of OCEs in each family. Fourty four OCE folds were detected in Leotiomycetes, Sordariomycetes and Dothideomycetes, with only five OCE folds were restricted to a single lineage. Folds enriched in the Sordariomycetes included MAX, Alpha Toxin and Tox3, folds enriched in the Leotiomycetes were RALPH, Blugra1, and DotL, and folds enriched in the Dothideomycetes were PAFB and ChitinaseA. No OCE fold was specific to mycoparasites, the Zuotin-like fold was the only one restricted to animal pathogens, 12 folds were restricted to plant pathogens including MAX and SIX3. Fourty-six OCE folds were detected in pathogens with mutualistic, necrotrophic and other pathogenic lifestyles.

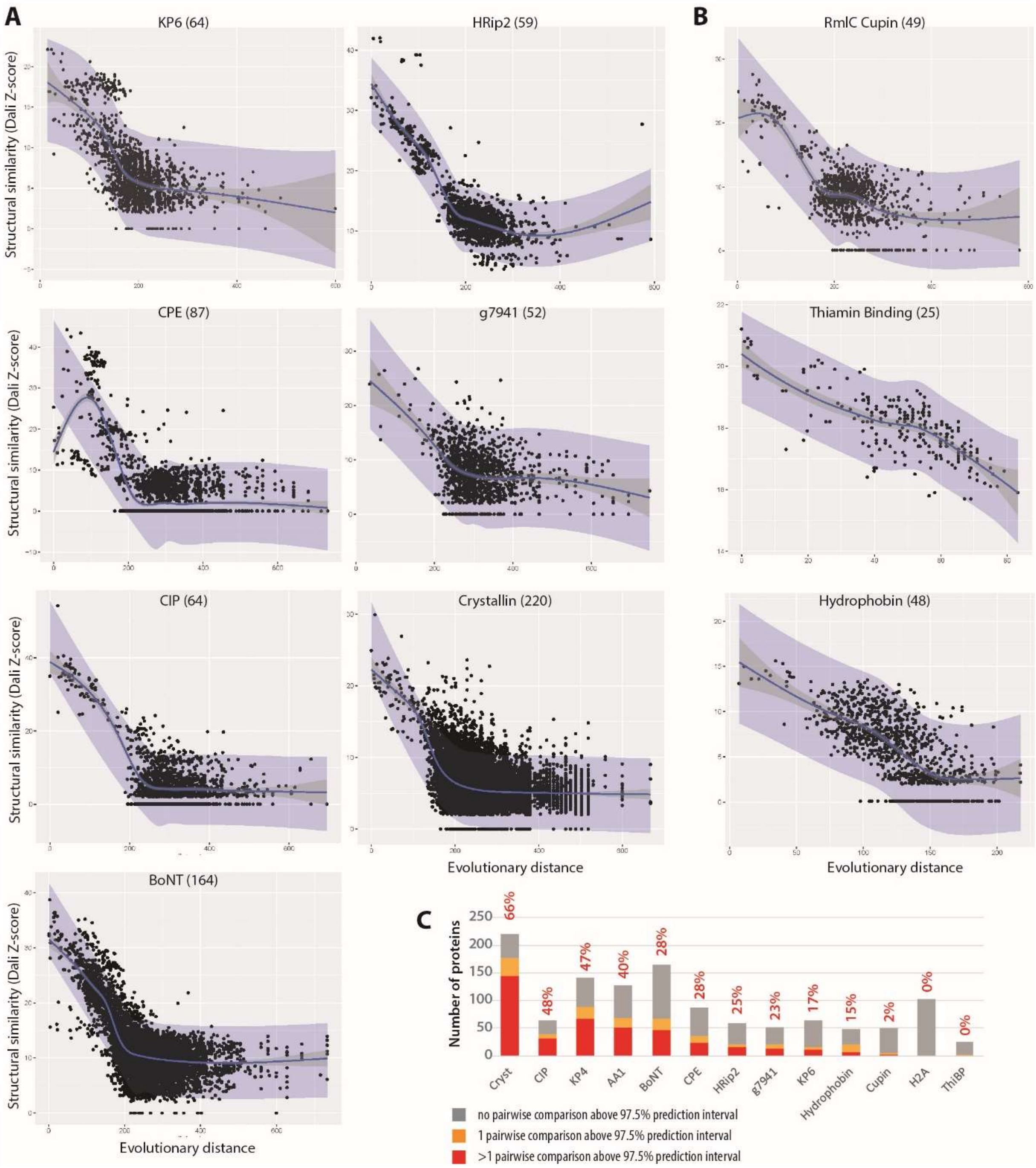

Supplementary Fig. 5. Relationship between structural similarity and sequence similarity in OCEs. Structural similarity (Dali Z-score) according to evolutionary distance (Jukes-Cantor corrected distance calculated on curated sequence alignments) for seven OCE groups **(A)** and three housekeeping control groups **(B)**. The blue lines show local average. We focused on pairwise protein comparisons above the 97.5% prediction interval as remarkable for highlighting proteins with high structural similarity in spite of significant sequence divergence. **(C)** Number of proteins with 0, 1 or more than 1 pairwise comparison above the 97.5% prediction interval. The percentage of proteins with 1 or more comparison above the 97.5% prediction interval is indicated above each bar.

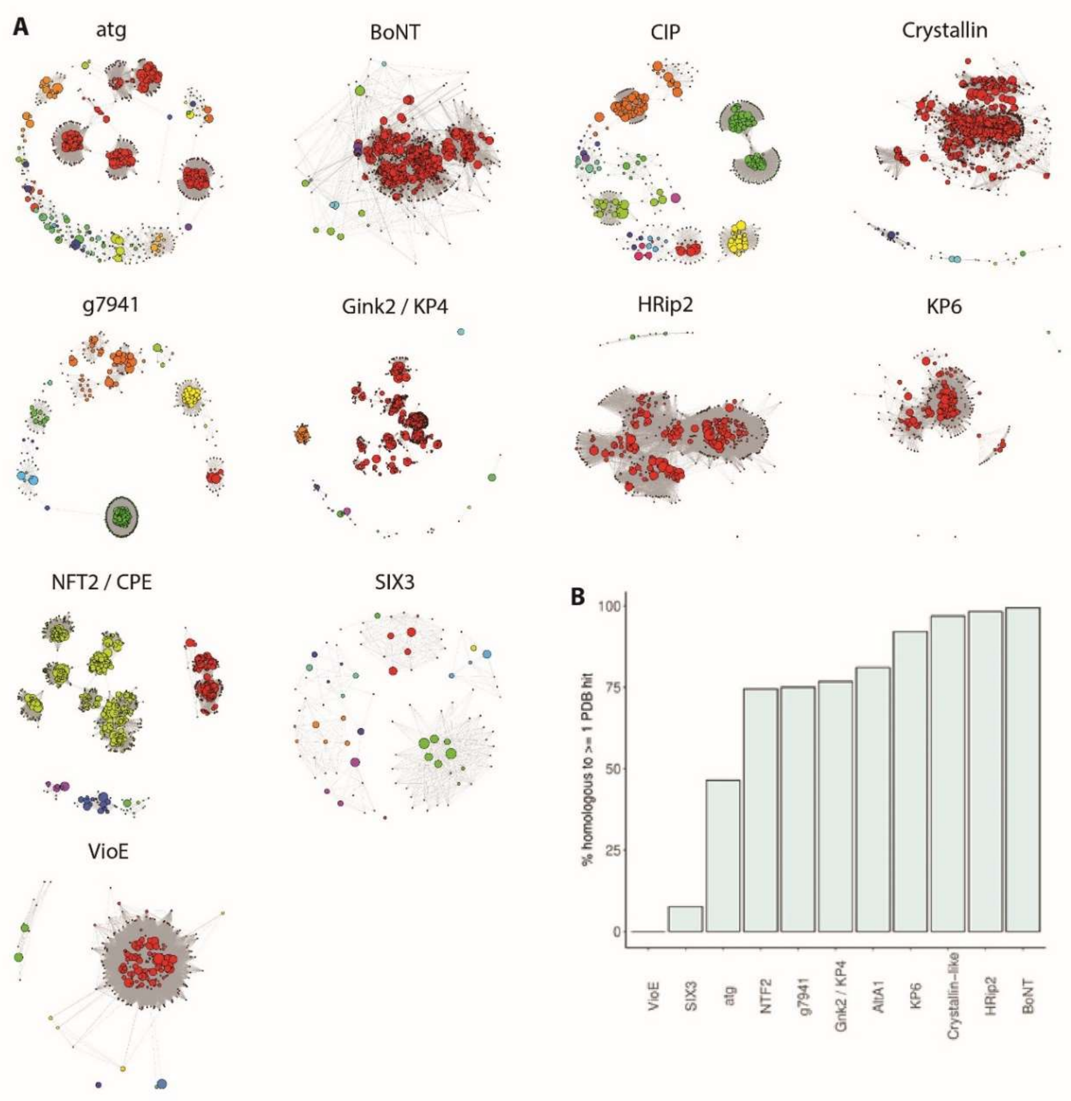

Supplementary Fig. 6. Profile HMM-HMM comparisons demonstrate deep ancestry of most members of OCE structural families. (A) We used BLASTp to identify up to 1,000 homologues from the NCBI nr database fungal division of each of the OCEs across 12 of the largest OCE clusters, including AltA1, atg, BoNT, CIP, Crystallin, g7941, Gink2 / KP4, HRip2, KP6, NTF2 / CPE, SIX3 and VioE. We identified 593,597 BLASTp hits to these OCE families and grouped them into clusters with >= 80 % coverage of all members and a similarity of >= 30 %. This generated 3,467 clusters made up of 590,032 proteins and 3,565 singletons that did not meet these alignment criteria. We then conducted all vs all pairwise HMM-HMM comparisons between clusters and sequence-HMM comparisons between singletons and clusters for each of the families. Each circle represents a different MMseqs cluster and each super-cluster is represented with a different colour. Super-clusters were identified using all v all HMM comparisons between MMseqs clusters. Nodes are connected where there is a significant HMM match between them. **(B)** Using the same approach, and additionally incorporating the amino acid sequences of the predicted OCEs, PDB structural hits from fungi, and the top 1,000 BLASTp hits of PDB structural hits, we found that for 8/11 families tested, >= 75 % of OCEs had evidence of remote homology to their PDB structural hits. For 4/11 families >= 90 % of OCEs had remote homology to their PDB structural hits. Only one family, VioE, contained no members with remote homology to at least one structural hit from PDB.

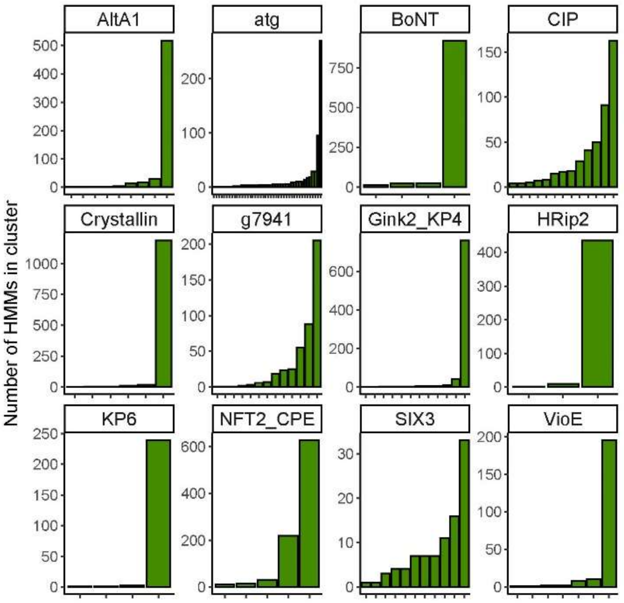

Supplementary Fig. 7. Identification of super-clusters of potentially evolutionary-related OCEs. All super-clusters for each family are laid out on the x axis from smallest to largest. The y axis shows the number of MMseqs clusters that make up the super-cluster. We defined super-clusters of potentially evolutionarily related sequences as groups of HMMs / singletons where each member was homologous to at least one other member in the group. We found that homologues of eight of the 12 families grouped into a major ancestral super-cluster that comprised at least 60 % of the HMMs and singletons. For instance, the largest super-cluster was found for the Crystallin fold, and it comprised 1,187 (96 %) HMMs and singletons. The second largest Crystallin fold HMM super-cluster only contained 19 HMMs and singletons. For the analysis of the Alt-A1 family, we also included the homologues of the top five PDB hits of all OCEs, which included many metazoan and bacterial proteins. The 11,251 AltA1 homologues formed 7 super-clusters with possible common ancestry. Super-cluster 1, was present in 10 divergent fungal classes spanning Basidiomycetes and Ascomycetes, and it contained 627 related HMMs / singletons spanning 5,135 proteins. This super-cluster contained the PDB entries for the structurally similar proteins TSP1, AltA1 and PevD1. Super-cluster 2, which contained 44 HMMs / singletons and 220 proteins, spanned four Ascomycete and one Basidiomycete class, whereas super-cluster 6 contained one HMM and one singleton, together comprising 28 proteins, and was only present in the Leotiomycetes. Overall, HMM comparisons suggest that the majority of effector folds share distant common ancestry. However, we cannot rule out convergent *de novo* emergence of similar effector folds, or fold recruitment from unrelated sequences, especially since several of the families formed distinct clusters with no detectable homology based on HMM-HMM comparisons.

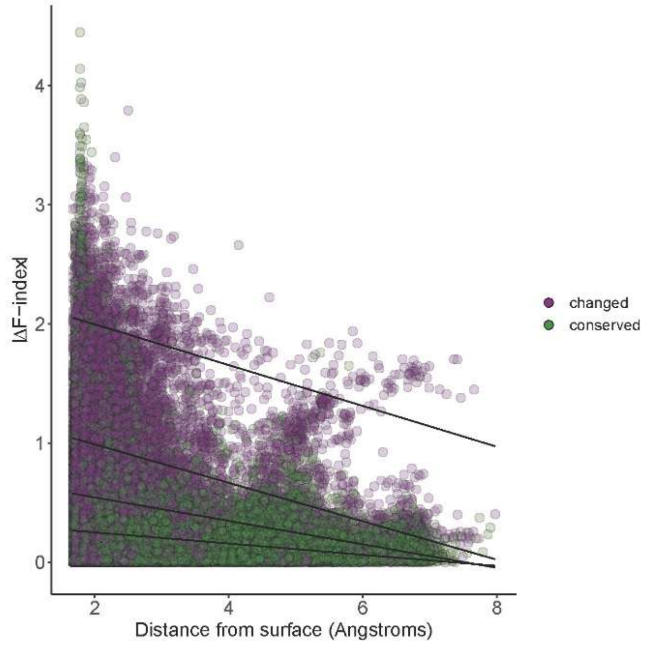

Supplementary Fig. 8. Relationship between residues frustration variation (y-axis) and surface exposure (x-axis) in three OCE families. Conserved residues are shown in green, variants in purple. 0.5, 0.75, 0.9 and 0.99 quantile regression lines are shown in black. To test whether amino-acids at the surface vary in frustration during evolution more than buried residues, we aligned each descendant with their respective N0 ancestor and calculated absolute delta frustration index for each residue. We then tested the correlation between average atomic distance from the surface in Angstroms and absolute change in frustration. For this, distance to protein surface was calculated using the MSMS 2.6.1 software from the ssbio python package[6]. Although there was a weak negative correlation between distance from surface and delta frustration index (Spearman's rho = 0.2, P = 0), there was a much greater variability in delta frustration index among amino acids at the surface of the protein compared with in the core. There was also an overall increase in delta frustration per residue among surface exposed residues (< 2.5 angstroms distance from the surface on average) relative to buried residues. Overall, OCE residues close to the surface have changed frustration patterns more during evolution than residues that are buried.

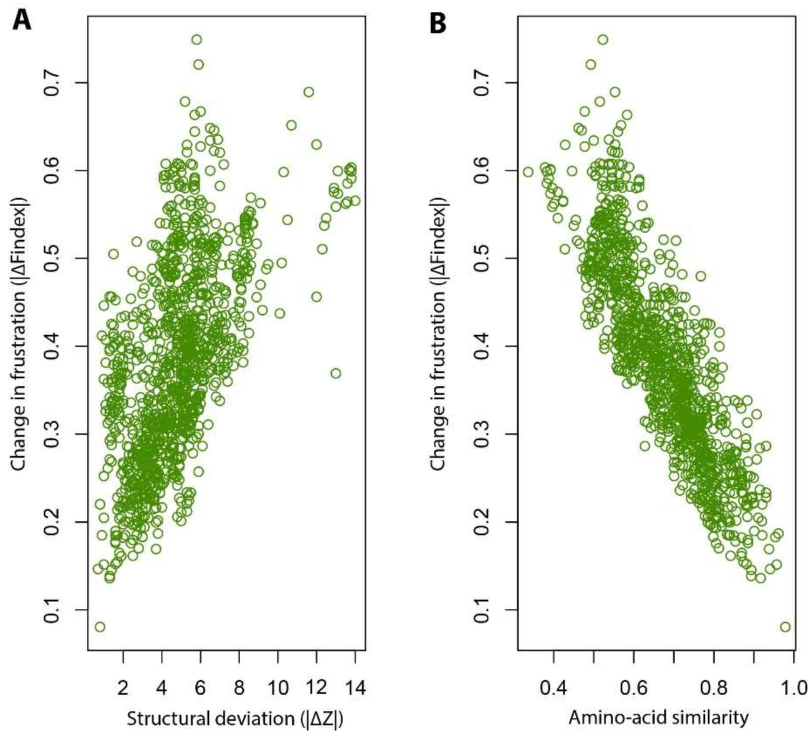

Supplementary Fig. 9. Relationship between absolute change in frustration for any given site relative to the ancestor (Y-axis) and structural deviation from common ancestor (A) and calculated sequence similarity between ancestors and their descendant OCEs (B). To study the relationship between change in frustration and change in structure in OCEs, we compared all OCEs against their common ancestor at the root of the tree and subtracted Z scores from the ancestor self-match Z score to estimate structural deviation. Next, we calculated the average absolute change in frustration for any given site relative to the ancestor, and calculated sequence similarity between ancestors and their descendant OCEs. There was a significant correlation between absolute change in structure and absolute change in frustration (on average per residue) (rho = 0.59, P = 0) (**A**) and a stronger anticorrelation between sequence similarity to the ancestor and absolute change in frustration (rho = 0.87, P = 0) (**B**). This indicates that mutation is a major driver of frustration change in OCEs and that frustration variation (increase and decrease in frustration altogether) has a strong impact on structure of OCEs.

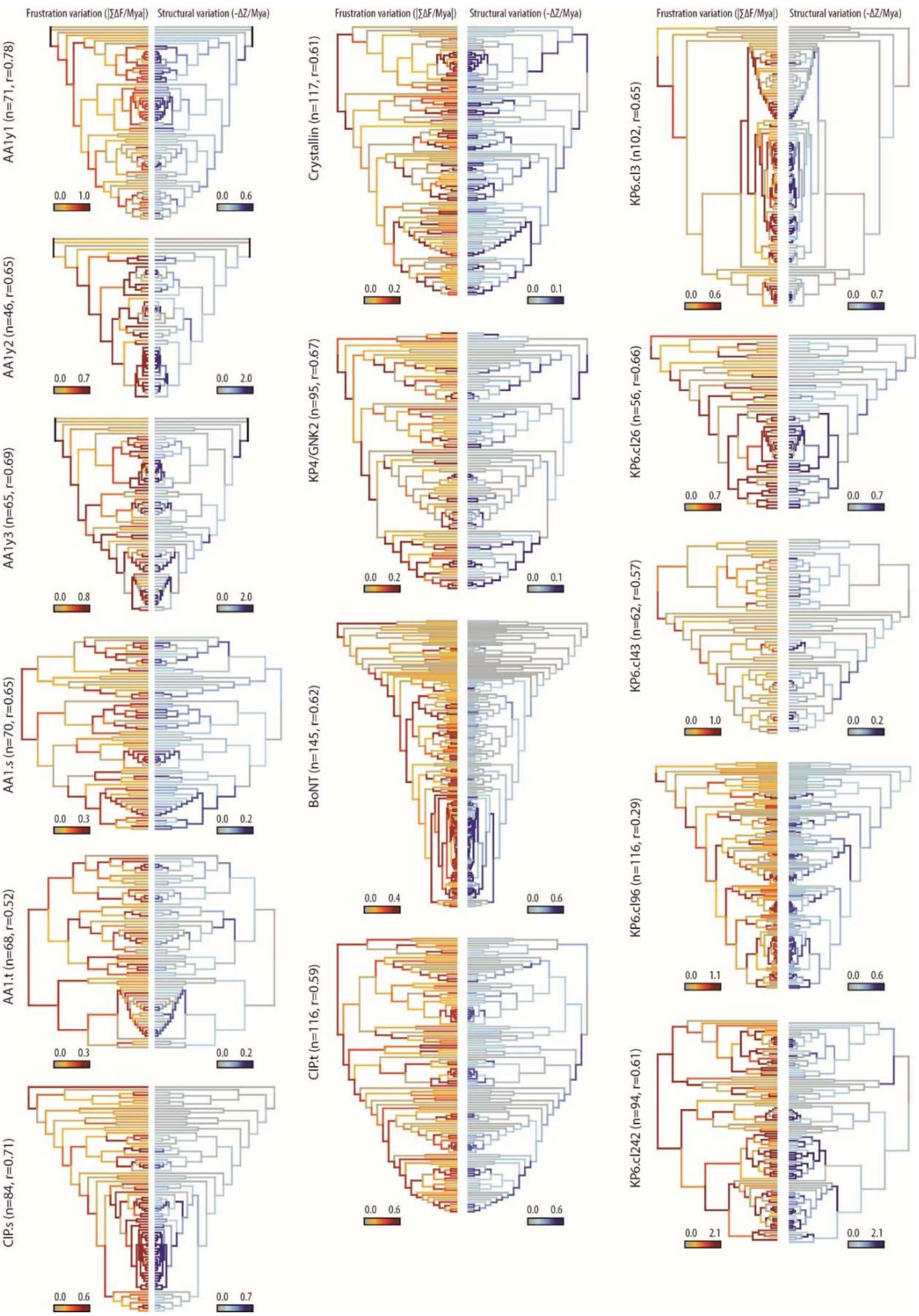

Supplementary Fig. 10. Frustration (red, left side) and structural (blue, right side) variation during the evolution of 15 OCE clusters from 6 families mapped on time-calibrated phylogenies. N is the number of modern OCEs per group, r is Pearson's product-moment correlation between frustration and structural variation across all branches of the tree. Mya, Million years ago. The distribution of frustration and structure variation along phylogenetic trees indicated that these parameters fluctuated over the evolution of OCEs.

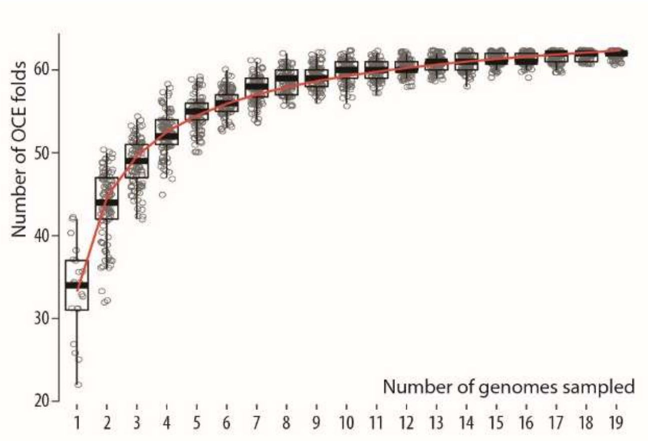

Supplementary Fig. 11. Logarithmic regression on the number of distinct OCE folds detected according to the number of fungal genomes sampled. In order to estimate the total number of OCE folds in the fungal kingdom, we randomly sampled 1 to 19 fungal genomes and counted the number of OCE folds detected. We then used a logarithmic regression on the number of OCE folds detected according to the number of fungal genomes sampled. Assuming our sampling of fungal species is representative of the actual diversity, we would expect a maximum of 70 OCE folds to exist across 2 million fungal species. The red line shows logarithmic regression. Dots show the number of distinct OCEs (out of the 62 from the complete dataset) in 100 random samples of the 20 fungal genomes. Boxplots show median values (thick line), first and third quartile values (box) and 1.5 times the interquartile range (whiskers).

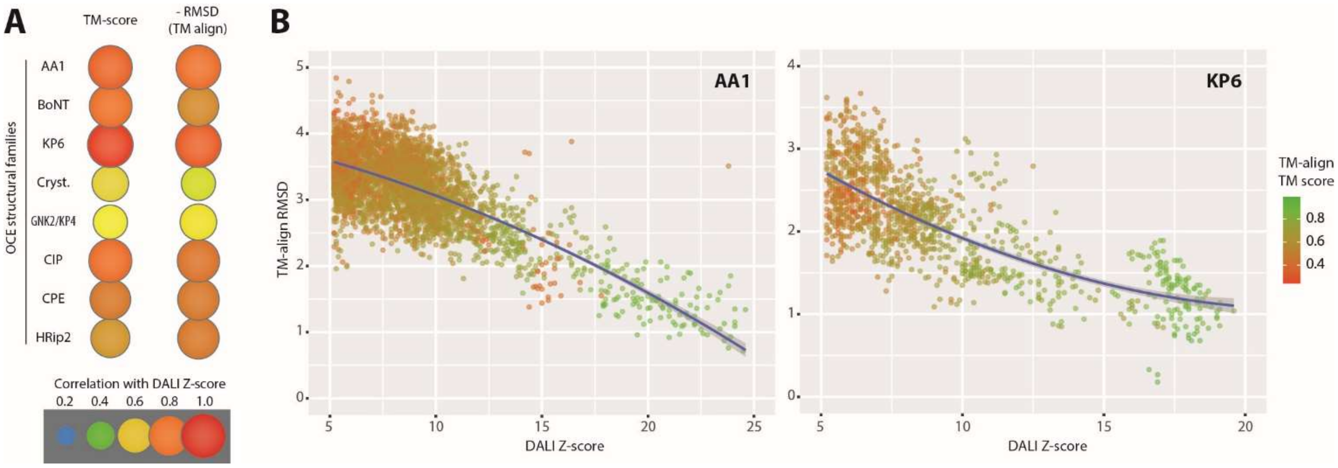

Supplementary Fig. 12. Correlation between structural similarity metrics in the major OCE families analyzed in our work. (A) Pearson correlation coefficient between DALI Z-score and TM-score or RMSD calculated by TM-align for the 8 major OCE families analyzed in our manuscript. (B) Relationship between DALI Z-score, RMSD and TM-score in the two OCE families most extensively analyzed in our manuscript (AA1 and KP6). Blue line show LOESS regression, grey ribbons show 90% confidence intervals.

SUPPLEMENTARY REFERENCES

1. Pennington, H. G. *et al.* The fungal ribonuclease-like effector protein CSEP0064/BEC1054 represses plant immunity and interferes with degradation of host ribosomal RNA. *PLoS Pathog.* **15**, e1007620 (2019).
2. Gupta, G. D., Bansal, R., Mistry, H., Pandey, B. & Mukherjee, P. K. Structure-function analysis reveals Trichoderma virens Tsp1 to be a novel fungal effector protein modulating plant defence. *Int. J. Biol. Macromol.* **191**, 267–276 (2021).
3. Li, N. *et al.* Structure of Ustilago maydis killer toxin KP6 α-subunit. A multimeric assembly with a central pore. *J. Biol. Chem.* **274**, 20425–20431 (1999).
4. De la Concepcion, J. C. *et al.* Polymorphic residues in rice NLRs expand binding and response to effectors of the blast pathogen. *Nat. Plants* **4**, 576–585 (2018).
5. Liu, M. *et al.* Crystal Structure Analysis and the Identification of Distinctive Functional Regions of the Protein Elicitor Mohrip2. *Front. Plant Sci.* **7**, 1103 (2016).
6. Mih, N. *et al.* ssbio: a Python framework for structural systems biology. *Bioinformatics* **34**, 2155–2157 (2018).
