# Supplementary material for: Surface frustration re-patterning underlies the structural landscape and evolvability of fungal orphan candidate effectors
Source: Nat Commun. 2023 Aug 28;14:5244. doi: 10.1038/s41467-023-40949-9 (PMC10462633; doi:10.1038/s41467-023-40949-9)
Supplement: Supplementary file 3 — Description of Additional Supplementary Files [file 41467_2023_40949_MOESM3_ESM.pdf]

Description of Additional Supplementary Files:

File name: Supplementary Movie 1

Description: Videos illustrating the mapping of relative surface exposure and structural variability in Alt-A1 group. Color scales are as in Figure 2 (.mp4 format).

File name: Supplementary Movie 2

Description: Videos illustrating the mapping of relative surface exposure and structural variability in BoNT group. Color scales are as in Figure 2 (.mp4 format).

File name: Supplementary Movie 3

Description: Videos illustrating the mapping of amino-acids conservation on KP6 cluster 43 ancestor. Color scales are as in Figure 3 (.mp4 format)

File name: Supplementary Movie 4

Description: Videos illustrating the mapping of amino-acids conservation on Alt-A1 clade 25 ancestor. Color scales are as in Figure 3 (.mp4 format)

File name: Supplementary Movie 5

Description: Videos illustrating the mapping of co-selected mutation patches on KP6 cluster 43 ancestor. Color scales are as in Figure 3 (.mp4 format)

File name: Supplementary Movie 6

Description: Videos illustrating the mapping of co-selected mutation patches on Alt-A1 clade 25 ancestor. Color scales are as in Figure 3 (.mp4 format)

File name: Supplementary Movie 7

Description: Videos illustrating the mapping of residue net stabilization effects on KP6 cluster 43 ancestor. Color scales are as in Figure 3 (.mp4 format)

File name: Supplementary Movie 8

Description: Videos illustrating the mapping of residue net stabilization effects Alt-A1 clade 25 ancestor. Color scales are as in Figure 3 (.mp4 format)
