# Supplementary material for: Surface frustration re-patterning underlies the structural landscape and evolvability of fungal orphan candidate effectors
Source: Nat Commun. 2023 Aug 28;14:5244. doi: 10.1038/s41467-023-40949-9 (PMC10462633; doi:10.1038/s41467-023-40949-9)
Supplement: Supplementary file 12 — Reporting Summary [file 41467_2023_40949_MOESM12_ESM.pdf]

nature portfolio

Corresponding author(s): Sylvain Raffaele

Last updated by author(s): Jul 19, 2023

Reporting Summary

Nature Portfolio wishes to improve the reproducibility of the work that we publish. This form provides structure for consistency and transparency in reporting. For further information on Nature Portfolio policies, see our Editorial Policies and the Editorial Policy Checklist.

Statistics

For all statistical analyses, confirm that the following items are present in the figure legend, table legend, main text, or Methods section.

n/a	Confirmed	
☐	☒	The exact sample size (*n*) for each experimental group/condition, given as a discrete number and unit of measurement
☐	☒	A statement on whether measurements were taken from distinct samples or whether the same sample was measured repeatedly
☐	☒	The statistical test(s) used AND whether they are one- or two-sided<br>*Only common tests should be described solely by name; describe more complex techniques in the Methods section.*
☐	☒	A description of all covariates tested
☐	☒	A description of any assumptions or corrections, such as tests of normality and adjustment for multiple comparisons
☐	☒	A full description of the statistical parameters including central tendency (e.g. means) or other basic estimates (e.g. regression coefficient) AND variation (e.g. standard deviation) or associated estimates of uncertainty (e.g. confidence intervals)
☐	☒	For null hypothesis testing, the test statistic (e.g. *F*, *t*, *r*) with confidence intervals, effect sizes, degrees of freedom and *P* value noted<br>*Give P values as exact values whenever suitable.*
☒	☐	For Bayesian analysis, information on the choice of priors and Markov chain Monte Carlo settings
☒	☐	For hierarchical and complex designs, identification of the appropriate level for tests and full reporting of outcomes
☒	☐	Estimates of effect sizes (e.g. Cohen's *d*, Pearson's *r*), indicating how they were calculated

Our web collection on statistics for biologists contains articles on many of the points above.

Software and code

Policy information about availability of computer code

Data collection

No software was used for data collection

Data analysis

Mature sequences of secreted proteins were searched for conserved domains with the hmmscan.pl script using the Pfam-A 35.0 database
Secreted proteins were predicted using signalP-4.1g (initial screen) and signalP6.0 (evolutionary analyses)
Orphans were searched for intrinsic disorder regions using the EspritZ server version 1.3
OCE structures were predicted using the ColabFold: AlphaFold2 w/ MMseqs2 BATCH
Structural similarity between was calculated using DALILite 5.0
Similarity to known protein structures was assessed using DALI against a local instance of the PDB database as accessed on May 12, 2022
Matrix of pairwise similarity Z-scores was converted into a structural similarity network with the igraph package in RStudio v1.4.1106
Network was exported as a .gml file using the plotCytoscapeGML function from the NetPathMiner v1.8.0 package
Structural similarity was also assessed using TM-align v. 2022/4/12
Multiple alignments were generated with muscle
Evolutionary distance was calculated with the distmat program in EMBOSS
Neighbor-joining trees were created with BioNJ
Structures were aligned in UCSF Chimera version 1.11.2 build 41376
HMM clusters were generated with Decipher version 2.24.0 and hhsuite3 version 3.3.0
sequences were clustered using MMseqs2 version 13.45111
Ancestral OCE sequences were determined with GRASP version 2020.05.05
Redundant sequences were filtered out with Cdhit
Phylogenetic trees were built using phyML 3.1
Time-calibration used the chronos function of the R package ape v. 5.6-2 and TimeTree 5

Residue frustration was calculated using frustratometeR version 0.1.0
Trees were rendered using R functions from the package phytools

For manuscripts utilizing custom algorithms or software that are central to the research but not yet described in published literature, software must be made available to editors and reviewers. We strongly encourage code deposition in a community repository (e.g. GitHub). See the Nature Portfolio guidelines for submitting code & software for further information.

Data

Policy information about availability of data

All manuscripts must include a data availability statement. This statement should provide the following information, where applicable:

- Accession codes, unique identifiers, or web links for publicly available datasets
- A description of any restrictions on data availability
- For clinical datasets or third party data, please ensure that the statement adheres to our policy

Source data are provided with this paper, and on zenodo.org under doi: 10.5281/zenodo.7506581. Complete predicted proteomes for 20 Ascomycete fungi were downloaded from the public repositories listed in Source Data as follows:
https://genome.jgi.doe.gov/portal/Cenge3/Cenge3.download.html;
https://fungi.ensembl.org/Zymoseptoria_tritici_st99ch_3d7_gca_900091695/Info/Index
https://genome.jgi.doe.gov/portal/Alalte1/Alalte1.download.html
https://genome.jgi.doe.gov/portal/Lepmu1/Lepmu1.download.html
https://genome.jgi.doe.gov/portal/Ampqui1/Ampqui1.download.html
https://genome.jgi.doe.gov/portal/Blugr2/Blugr2.download.html
https://genome.jgi.doe.gov/portal/Oidma1/Oidma1.download.html
https://genome.jgi.doe.gov/portal/Pseudest1/Pseudest1.download.html
http://fungi.ensembl.org/Botrytis_cinerea/Info/Index
https://www.ncbi.nlm.nih.gov/genome/?term=PRJEB36718
https://www.ncbi.nlm.nih.gov/genome/?term=PRJNA348385
https://www.ncbi.nlm.nih.gov/genome/?term=PRJEB36746
https://genome.jgi.doe.gov/portal/FoxFo5176/FoxFo5176.download.html
https://www.ncbi.nlm.nih.gov/genome/?term=MBGI00000000
https://genome.jgi.doe.gov/portal/Dreco1/Dreco1.download.html
https://fungi.ensembl.org/Claviceps_purpurea_20_1_gca_000347355/Info/Index
https://www.ncbi.nlm.nih.gov/genome/?term=PRJNA431450
https://genome.jgi.doe.gov/portal/Metro1/Metro1.download.html
https://genome.jgi.doe.gov/portal/Magor1/Magor1.download.html
http://fungi.ensembl.org/Verticillium_dahliaejr2/Info/Index
The following databases were used:
Proteins were searched for conserved domains Pfam-A 35.0 database
Similarity to known protein structures was assessed using DALI against a local instance of the PDB database as accessed on May 12, 2022
Time-calibration of phylogenies used the TimeTree 5 database

Human research participants

Policy information about studies involving human research participants and Sex and Gender in Research.

Reporting on sex and gender	This study does not involve human research
Population characteristics	This study does not involve human research
Recruitment	This study does not involve human research
Ethics oversight	This study does not involve human research

Note that full information on the approval of the study protocol must also be provided in the manuscript.

Field-specific reporting

Please select the one below that is the best fit for your research. If you are not sure, read the appropriate sections before making your selection.

☒ Life sciences ☐ Behavioural & social sciences ☐ Ecological, evolutionary & environmental sciences

For a reference copy of the document with all sections, see nature.com/documents/nr-reporting-summary-flat.pdf

Life sciences study design

All studies must disclose on these points even when the disclosure is negative.

Sample size	Twenty fungal species were chosen to cover the phylogenetic and lifestyle diversity of the Pezizomycotina. This was set as the minimum

Sample size	number to include all major lineages and lifestyles in this group.<br>Their complete proteomes or complete structural families were analyzed when relevant.<br>Null probability of co-mutation occurrence was determined by shuffling extant variable residues 10,000 times. P-values were calculated using Bonferroni correction for multiple testing.<br>For mutational scans, all positions were mutated to alanines or deleted, sets of 100 random sequences with multiple mutations were analyzed to reach a sample size comparable to modern, alanine scan and deletion scan sets.<br>No sample size calculation was performed.
Data exclusions	For structural landscape analyses, proteins were selected based on (i) a predicted secretion signal, (ii) mature size shorter than 300 amino-acids, (iii) absence of recognized PFAM protein domains, (iv) intrinsic disordered regions covering less than 50% of the sequence, (v) pLDDT score >50; (vi) at least three analog structures at Z>= 5.2. These criterion allowed focusing on orphan candidate effectors and reducing the inclusion of spurrious structures.
Replication	The findings were assessed across the complete repertoire of orphan effector candidates with no exclusion. For family-wise findings, the results from at least two unrelated families spanning >15 species and >65 proteins are reported. Analyses reported in the manuscript were successfully replicated on all protein sets, with statistics reported in the Source Data File.
Randomization	Effector families were determined based on structural similarity. Random multiple mutants were designed by assigning randomly amino-acids independently to each position in proteins.
Blinding	Association was determined between protein properties. Other blind designs are not applicable to this study in which protein sequences and structures are the primary analysis material.

Reporting for specific materials, systems and methods

We require information from authors about some types of materials, experimental systems and methods used in many studies. Here, indicate whether each material, system or method listed is relevant to your study. If you are not sure if a list item applies to your research, read the appropriate section before selecting a response.

Materials & experimental systems

n/a	Involved in the study
☒	☐ Antibodies
☒	☐ Eukaryotic cell lines
☒	☐ Palaeontology and archaeology
☒	☐ Animals and other organisms
☒	☐ Clinical data
☒	☐ Dual use research of concern

Methods

n/a	Involved in the study
☒	☐ ChIP-seq
☒	☐ Flow cytometry
☒	☐ MRI-based neuroimaging
